# Landscape Use and Co-Occurrence Patterns of Neotropical Spotted Cats

**DOI:** 10.1371/journal.pone.0168441

**Published:** 2017-01-04

**Authors:** Mariana B. Nagy-Reis, James D. Nichols, Adriano G. Chiarello, Milton Cezar Ribeiro, Eleonore Z. F. Setz

**Affiliations:** 1 Department of Animal Biology, Universidade Estadual de Campinas (UNICAMP), Campinas, São Paulo, Brazil; 2 Patuxent Wildlife Research Center, U.S. Geological Survey, Laurel, Maryland, United States of America; 3 Department of Biology, Universidade de São Paulo (USP), Ribeirão Preto, São Paulo, Brazil; 4 Spatial Ecology and Conservation lab (LEEC), Department of Ecology, Universidade Estadual de São Paulo (UNESP), Rio Claro, São Paulo, Brazil; Universidade de Aveiro, PORTUGAL

## Abstract

Small felids influence ecosystem dynamics through prey and plant population changes. Although most of these species are threatened, they are accorded one of the lowest research efforts of all felids, and we lack basic information about them. Many felids occur in sympatry, where intraguild competition is frequent. Therefore, assessing the role of interspecific interactions along with the relative importance of landscape characteristics is necessary to understand how these species co-occur in space. Here, we selected three morphologically similar and closely related species of small Neotropical cats to evaluate the roles of interspecific interactions, geomorphometry, environmental, and anthropogenic landscape characteristics on their habitat use. We collected data with camera trapping and scat sampling in a large protected Atlantic forest remnant (35,000 ha). Throughout occupancy modeling we investigated whether these species occur together more or less frequently than would be expected by chance, while dealing with imperfect detection and incorporating possible habitat preferences into the models. We used occupancy as a measure of their habitat use. Although intraguild competition can be an important determinant of carnivore assemblages, in our system, we did not find evidence that one species affects the habitat use of the other. Evidence suggested that proximity to the nature reserve (a more protected area) was a more important driver of Neotropical spotted cats’ occurrence than interspecific interactions or geomorphometry and environmental landscape characteristics—even though our entire study area is under some type of protection. This suggests that small felids can be sensitive to the area protection status, emphasizing the importance of maintaining and creating reserves and other areas with elevated protection for the proper management and conservation of the group.

## Introduction

Predators play an important role in regulating ecosystem functioning and dynamics [1, 2]. They affect prey populations and, as a result, alter plant abundance, composition, succession, dispersion, and diversity [2, 3]. Consequently, the loss of this key group can lead to regime shifts, alternative states of ecosystems, and possible losses of ecosystem services [2]. Given the role of carnivores on ecosystem functioning and their sensitivity to the environment, landscapes with these animals—implying a relatively intact food web—have high potential for ecological integrity [4], which encompasses ecosystem health, biodiversity, stability, and sustainability [5]. Therefore, maintenance of carnivores can serve as a useful tool for protected area design and conservation planning [6].

Among the carnivores, felids are extreme as obligatory flesh eaters [7]. Historically, felids have suffered several anthropogenic impacts, which represent a major cause of felid mortality in several regions of the globe and account for up to 70% of deaths in some populations [8, 9]. For example, larger felids are frequently involved in conflicts with humans due to preying on domestic animals or livestock [9–13], and small felids are constantly suffering from fur trade [8, 14]. Although several countries banned the export of wildcat skins and signed the Convention on International Trade in Endangered Species of Wild Flora and Fauna (CITES) [9], illegal hunting of felids still occurs (e.g., [11]). Felids can also be exposed to diseases carried by domestic carnivores and to poaching, which even at moderate levels over a relatively short period of time can lead to massive population decline [9, 15, 16]. Hunting and these other human-related pressures are most likely to occur in areas with high accessibility or low (or inefficient) protection status, and in unrestricted areas surrounding protected areas [9, 17–19]. In highly protected populations, anthropogenic mortality is rare [9]. More recently, mammalian carnivores face local extinction due to habitat loss and fragmentation, exacerbated by their relatively large home ranges, low densities, and direct persecution by humans [4, 20–22].

Given the current scenario, understanding how species relate spatially to the environment and to human disturbances is critical to the assignment of areas for conservation and the development of conservation strategies [23–26]. Besides landscape characteristics, interspecific interactions may also regulate the occurrence, distribution or persistence of species [27]. In carnivore assemblages, intraguild interference competition and killing are potentially important determinants of species abundance and distribution and can lead to adaptive responses in use of space and activity patterns [7, 28, 29], which enable the coexistence of morphologically similar species [30].

Because of relatively recent divergence and constraints imposed by foraging and diet, felids present similar morphologies [31, 32]. Thus, they are a good model to understand how closely related and morphologically similar species can coexist. However, though felids are frequently sympatric, with the largest assemblages occurring in the tropical regions of the Americas, little attention has been given to the coexistence of these species and its implications [8]. The Neotropical spotted cats are the main sympatric small felids in Neotropical rainforests: *Leopardus pardalis—*ocelot, *L*. *wiedii*—margay, and *L*. *guttulus—*oncilla (formerly known as *L*. *tigrinus* [33]). These species have discrete differences in body sizes (8–15 kg, 3-9kg, and 1.5-3kg, respectively [34]) and share an important portion of their food sources [35, 36], therefore differential food exploitation is unlikely and intraguild competition is probably high [37]. They have been accorded one of the lowest research efforts of all felids, and more basic information on their biology and ecology is urgently needed [7, 8].

Frequently, studies on species occurrence and distribution assume that all species present at a location are detected with certainty. However, accounting for imperfect detection is fundamental to avoid omission errors (false absences) [38, 39] and resultant bias in parameter estimation [40]. Such omission errors may lead to incorrect inferences about species-habitat relationships or patterns of species co-occurrence [41]. A recent adaptation to occupancy models [42] allows such problems to be dealt with by incorporating detection probability, as well as possible habitat preferences, directly into the model set and evaluating co-occurrence patterns among pairs of species [41].

We investigated the role of geomorphometry, environmental and anthropogenic landscape attributes at multiple scales and interspecific interactions in the habitat use of Neotropical spotted cats in a large Atlantic forest remnant. Using a likelihood-based framework, we estimated the probability of occurrence and co-occurrence while accounting explicitly for imperfect detectability and habitat preferences. We used occupancy as a measure of habitat use, and developed specific models based on different hypotheses about effects of landscape characteristics and competition on the habitat use of Neotropical spotted cats. We have two alternative hypotheses: 1) The habitat use of Neotropical spotted cats is mainly driven by landscape characteristics—If landscape characteristics are important factors determining how spotted cats use the habitat, we would expect anthropogenic-related variables to be the main predictors of occurrence, given the sensitivity of carnivores to anthropogenic impacts [8, 9]. More specifically, because human-related pressures are most likely to occur in areas with low protection status or with high accessibility [9, 17–19], we would predict a negative association between habitat use and road density (a measure of human accessibility) and between habitat use and distance to a more protected area (a reserve). We would also expect that prey, hydrographic density, and forest cover would have a positive effect on habitat use, since those variables represent important resources (food and water availability) and high-quality habitats, but they would have a smaller influence than the human-related variables. We would also expect elevation to have a weak influence on how Neotropical spotted cats use the habitat, unless species are segregating in altitude due to the montaneous terrain in our study area; this phenomenon has been observed in other taxa (e.g., [41]). 2) The habitat use of Neotropical spotted cats is mainly driven by interspecific interactions—If competition is a major determinant of the habitat use of Neotropical spotted cats, we would expect their occupancy and/or detection probability to be lower when another spotted cat is present or detected; co-occurrence should be less than expected by chance, predicting avoidance, considering the commonness of interference intraguild competition and killing among carnivores [7, 28, 29]. We believe that knowledge of these potential relationships will be useful for planning management actions towards the conservation of this key group in Neotropical forests and helpful in clarifying the role of interspecific interactions on the occurrence of small felids, which could benefit our understanding of how small felids occur in other ecosystems worldwide.

## Methods

### Study area

**Fig 1:**
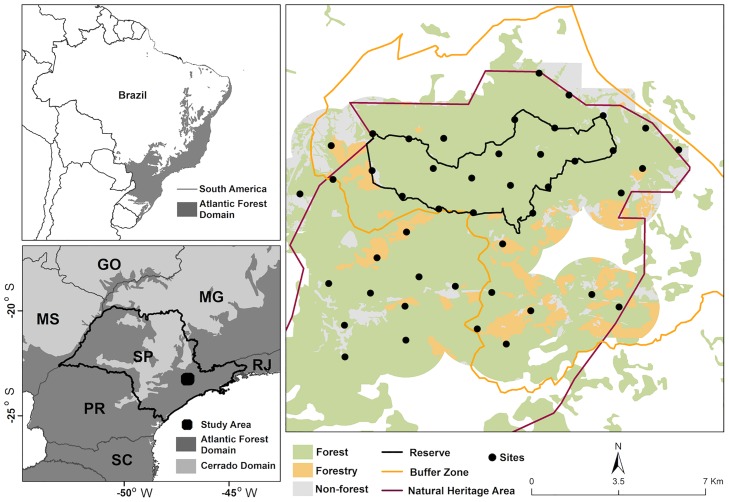
Study area. Study area and the sampling sites’ locations at Serra do Japi (Brazil) where Neotropical spotted cats were sampled using camera trap and scat sampling.

Serra do Japi (southeast Brazil, coordinates 47°03'40"W to 46°52'20"W and 23°22'30"S to 23°11'35"S; Fig 1) is one of the few large remnants of Atlantic Forest. The Atlantic Forest represents a global hotspot for biodiversity conservation [43], and currently it is highly fragmented (more than 80% of the remnants are < 50 ha in size), highly isolated (average distance between fragments is 1,440 m), and under negative edge influences (73% of remnants are 250 m from any forest edge) [44]. The study area is a Natural Heritage Area (35,000 ha) considered part of the UNESCO’s Atlantic Forest Biosphere Reserve [45]. Located within this area is the Biological Municipal Reserve (REBIO—2,071 ha) surrounded by a Buffer Zone (11,946 ha) (Fig 1). The REBIO presents the highest protection status in the area, where the only permitted activities are research and education. In the Natural Heritage Area and in the Buffer Zone, however, agriculture, horticulture and other forms of economic activities are present. The vegetation of the area is characterized as semideciduous mesophilic forest with mountainous terrain and a seasonal climate [46]. The mean temperature is 19.7°C and the mean annual rainfall is 1,422 mm, with a dry and cold season occurring from April to September and a wet and warm season from October to March [46].

### Data collection

From April 2013 to September 2014, we collected data on three Neotropical spotted cats (*Leopardus pardalis*, *L*. *wiedii*, and *L*. *guttulus*) at 45 sampling sites (spaced approx. 1.5 km apart) distributed in a regular grid across the forest remnant. This distance between the sampling sites provided a good coverage of our study area, and it is in agreement with the TEAM “terrestrial vertebrate (camera trap) monitoring protocol implementation manual” [47]. Furthermore, we had no evidence for spatial autocorrelation in data while using this spacing (S1 Table).

We conducted three campaigns at each sampling site to collect data: 1- April 2013 to September 2013; 2- October 2013 to March 2014; 3- April 2014 to September 2014. A different group of 15 sampling sites was surveyed every two months within each campaign (see S1 Fig for details). Hence, 45 sites were sampled during each campaign. We used camera trapping (passive infrared camera traps; Bushnell Trophy Cam; N = 5,198 trap days) and scat sampling during the first and the second campaigns, and only scat sampling during the third campaign. All cameras were fixed about 20 cm above ground and no bait was used. Traps were installed with a minimum distance of approx. 50 m from roads or trails, and none of the sampling sites was located close to roads that were highly used or open to the public. We visited each sampling site six times and collected all scats found along a 1-km segment in the dirt road closest to each site. There was no overlap between neighboring sampled areas because each sampling site was usually located in the center of its 1-km segment.

In addition to the felids’ data, we collected information on their main prey using the records from the camera traps. This was possible due to the height at which the cameras were positioned and the number of records (i.e., detections: N_small mammals_ = 77; N_medium-sized mammals_ = 897; N_ground-dwelling birds_ = 1,936). Although cameras are more commonly used to survey medium- and large-sized mammals, they can also be used to collect data on small mammals [48–50] and ground-dwelling birds [51–53]. They may provide a new and cost-effective technique for surveying terrestrial small mammals, particularly when presence data are the main requirement of the survey [54–55]. The use of camera traps to collect data on small mammals to assess prey availability for carnivores was already performed [56], but here we went a step further and included imperfect detection on prey estimates (through occupancy modeling, see “Single-species occupancy models” section) instead of using the number of captures. We considered small-sized mammals (< 1 kg; mainly small rodents and small marsupials) and small birds (< 0.2 g; mainly passerines and doves) as the main prey for margay and oncilla, and small and medium-sized mammals (< 13 kg; mainly small rodents, small marsupials, opossums, Brazilian rabbit, paca, armadillo, and porcupine) as well as small and medium-sized birds (< 0.5 g; passerines, doves, and tinamous) for ocelot [35, 57–61].

### Identification of felids using tricology and genetics

Because felids defecate conspicuously to signal their presence [62, 63] and groom frequently [64], samples of scats with hairs are particularly easy to obtain. After washing the scats with running water and drying them, we collected the guard hairs found in each sample to identify the species to which the hair belonged. We cleaned the guard hairs with ethyl alcohol, and the cuticular impressions were obtained by pressing the hairs against a thin layer of nail varnish and leaving them to dry for three to five minutes on glass slides with the help of a bench vise (adapted from [65]). We photographed the cuticular impressions at 400x magnification (S2 Fig) and compared the pattern of the cuticles with our reference collection (obtained from hairs collected from museum specimens) and published guides [66, 67].

Hair sampling can lead to reliable detections of rare and cryptic animals [68], and the use of mammalian hair for identification of taxa, known as tricology, is an established low-cost method (e.g., [69–74]). This technique has also been proven to be as consistent as molecular methods for identification of some Neotropical felids [75]. To test the accuracy of our identification through tricology, we conducted molecular analysis for 74% of the samples (N = 49). We used mini-barcoding for molecular identification, comparing two markers from mitochondrial DNA [ATP6 (126 bp) and cytochrome oxidase I gene (COI) (187 bp)], applying the primers developed by [76]. The obtained sequences were compared with reference sequences from tissue samples of each species. We achieved confirmation for 100% of the samples, giving us confidence in our identification and confirming the reliability of the method.

### Landscape covariates

We mapped with Quantum Gis software [77] the vegetation cover, hydrography and roads of the study area using high resolution satellite image interpretation at a 1:5,000 scale and cartographic maps (Secretariat of Economy and Planning—São Paulo State Government, at 1:10,000). We validated the cover map by extensive field verification with a botanist (see acknowledgements).

Because observed responses of the organisms to the environment may depend on the extent at which the environment is being perceived (and thus measured; [21, 78, 79]), we adopted a multi-spatial extent—also found in the literature as multiple scales [78]–approach while testing the influence of the covariates on felid occupancy. The extents were defined as concentric circles (buffers) of 500 and 1,000 m radius around each sampling site, and we calculated each site covariate for each extent. The smaller buffer (which covers 78 ha) is equivalent to the minimum known size for an ocelot home range (see [80]). This extent is likely smaller than the average home range of the other two cats as well [80]. The area of the larger buffer (314 ha) is closer to the home range of some ocelots, but might be also regarded as spatially conservative, considering the huge variation in home range size known from this and the other two cats [80]. Further, the larger buffer is the maximum we can use without overlapping with buffers of adjacent sampling points.

We determined five landscape site covariates: mean elevation, percentage of high-quality forest cover, hydrographic density, road density, and ‘weighted distance to reserve border’. The percentage of high-quality forest cover was measured as the percentage of intermediate and advanced forest succession cover within each buffer, which was calculated using Geographical Resources Analysis Support System (GRASS) [81]. Road and hydrographic densities within each buffer were calculated with the Kernel density function in ArcGIS software [82]. We obtained the mean elevation at each buffer from digital elevation models (DEM) available from Topodata Geomorphic database of Brazil [83]. We measured the distance from each sampling site to the nearest boundary of the Biological Municipal Reserve (REBIO), giving negative distances (in meters) to sites within the REBIO and positive distances otherwise (i.e., the center of the reserve received the smallest value). Then the covariate ‘weighted distance to reserve border’ was obtained by multiplying these distances by the protection status weight of the subarea in which each sampling site was located (REBIO = 1; REBIO’s Buffer Zone = 2; within the Natural Heritage Area but outside these two areas = 3; outside these three areas = 4). For ocelot, we used the distance to reserve border instead of ‘weighted distance to reserve border’ (due to the lack of convergence in the models with this latter covariate). We normalized all covariates and used only covariates with low correlation (S2 Table) in the final model sets (i.e., group of models being tested together).

### Single-species occupancy models

We used occupancy modeling [42]—with a likelihood-based approach—to estimate the site occupancy (ψ) of each spotted cat species and their main potential prey, and to evaluate the influential factors on the felids’ occupancy while accounting for detection probability (*p*). Because the size of the home ranges of ocelots, margays, and oncillas likely exceed that of our sampling unit [84–87], we used occupancy as a measure of their habitat use instead of “true occupancy” [38, 88]. A basic assumption of the occupancy models is that the sites are closed to changes in occupancy during the repeated surveys [89]. This assumption can be relaxed when occupancy is interpreted as “use” and movement in and out of the sampled area is random [41, 89], similarly to our approach for the felids. We assessed the usage made of sites with various habitat characteristics within their home range, therefore we should interpret our result in a third-order selection level [90]. However, because individual animals were not identified, measures were made at the population level (i.e., it is a Design I study type; [91]).

We adopted a single-season approach [42], wherein data from all three campaigns were combined. Data from both methods (camera trapping and scat sampling) were included, but in different sampling occasions. The decision to adopt a single-season modeling instead of a multi-season approach was based on our previous analyses, which provided no evidence of changes in occupancy across the three campaigns. Specifically, we fit both full multi-season models and models where colonization and extinction were fixed to zero (no change), and the latter models were ranked as the best (S3 Table). We also developed models that included an effect of campaign/time on detection probabilities, but model selection results provided no evidence that this effect was needed (S4 Table).

We used a two-step approach while modeling the occupancy of each species of felid and prey: 1) assess spatial scale that best represents species’ response to each covariate; and 2) investigate sources of variation in ψ, determining the most influential covariates for occupancy, the parameter with biological meaning and in which we were most interested; as detailed next. To determine the scale that best represents each species’ response to the habitat, we used a general model for *p* (that consisted of as many potential covariates as possible) and allowed occupancy (ψ) to vary (following [92]) by only the focal habitat covariate measured at the extents of the two buffer sizes (S5 Table). After selecting the appropriate extent for each covariate, we developed another model set to investigate the variation in occupancy (S6 Table). In this second step, we allowed ψ to be constant (ψ(.)) or to vary as a function of either a single covariate or a combination of two (only additive effects). We built only models that translated plausible biological hypotheses regarding the effects of variables on each species’ occupancy (ψ). We used each covariate at its extent of stronger response for each species (from the previous step) and a general model for *p*. The potential covariates used in the general model for *p* were: method used to survey each sampling occasion (camera trapping or scat sampling), degree of soil coverage by plants or leaf litter on the roads where scats were sampled (0- no coverage; 1- low to medium coverage; and 2- high coverage), and percentage of high-quality forest cover at the 500 m buffer around each sampling site. By using a general model for the parameters that were not investigated within a specific model set, we reduced the possibility that imposed constraints (on *p*, for example) would result in residual sampling variation being attributed to a variation in occupancy.

We evaluated candidate models and estimated parameters using PRESENCE software [93] to determine the covariates that best explain occupancy. We ranked candidate models using the Akaike's Information Criterion adjusted for small sample size (AICc; N = number of sites) [94] and excluded all models that did not converge. We considered the covariate(s) from the top-ranked model(s) (i.e., models with ΔAICc<2) as the most likely determinant(s) of the species’ occupancy. When different spatial extents were equally plausible (ΔAICc<2), we chose to use the extent closer to the home range size of the spotted cats (1,000 m) in the final models. Additionally, we assessed the relative importance of each covariate by summing the Akaike weights (*w*_*i*_) of all the models (*i*) in which that covariate was present [94]. We applied model averaging [94] to estimate the overall occupancy of each felid at our study area and to estimate the site occupancy of each prey. The prey index was obtained by summing the site occupancy of all potential prey for each felid species.

### Co-occurrence models

We investigated whether the presence of one felid influences the occupancy and detection probability of another felid by pair-wise comparisons for all felid species using two-species single-season occupancy models [41]. We used the ψBa/*r*Ba parameterization in PRESENCE software [93], assuming that the dominant species was always the larger (i.e., greater mean body mass) in the analyzed pair (ocelot in ocelot-margay and ocelot-oncilla pairs, and margay in the margay-oncilla pair; [34]). The parameters estimated for occupancy were: ψA (occupancy of dominant species), ψBA (occupancy of subordinate species when the dominant species is present), and ψBa (occupancy of subordinate species when the dominant species is absent). We modeled ψA, ψBA, and ψBa, incorporating the best covariate revealed in the single-species models for each species to account for possible differences in habitat preferences. We also incorporated the method used to survey each sampling occasion (camera trapping and scat sampling) as a covariate for *p* in all co-occurrence analyses. We built models that assumed that the occupancy of the subordinate species was influenced by the dominant species (ψBA≠ψBa) or was independent of the dominant species (ψBA = ψBa).

For detection probability, the parameters estimated were: *r*A (probability of dominant species being detected when the subordinate species is present), *p*A (probability of dominant species being detected when the subordinate species is absent), *p*B (probability of subordinate species being detected when the dominant species is not present), *r*BA (probability of subordinate species being detected when the dominant species is present and detected), *r*Ba (probability of subordinate species being detected when the dominant species is present but not detected). We built models where the detection probability of the subordinate species was influenced by the presence (*p*B≠*r*Ba; *p*B≠*r*BA) or detection (*r*Ba≠*r*BA) of the dominant species or was independent of the dominant species (*p*B = *r*Ba = *r*BA), as well as models that assumed that the detection of the dominant species was influenced by the detection of the subordinate species (*r*A≠*p*A) or independent (*r*A = *p*A).

We calculated the species interaction factor (SIF) for occupancy (phi; [95]) and detection probability ($\text{delta }=\frac{r\text{A}\;\times \;r\text{BA}}{r\text{A}\;\times \;\left(\left(r\text{A}\;\times \;r\text{BA}\right)+\left(\left(1-r\text{A}\right)\;\times \;r\text{Ba}\right)\right)}$; adapted from the formula for phi from [95]). The SIF is a ratio of how likely the two species are to co-occur compared to what would be expected under a hypothesis of independence [94]. We obtained the parameter estimates by model averaging [95] the estimates of each species-pair model set (i.e., group of all models tested for each species-pair). If two species occur or are detected independently, SIF = 1. If SIF<1, species co-occur or are detected less frequently than would be expected if they were independent (i.e., avoidance). If SIF>1, species co-occur or are detected more frequently than expected (i.e., aggregation) [95].

To rank candidate models, we adopted the same procedure as the single-species analysis. To infer about the co-occurence patterns, we considered the estimated parameters (ψA, ψBA, ψBa, *r*A, *p*A, *p*B, *r*Ba, *r*BA), the relationships among them, the top-ranked model(s) (ΔAICc<2), and the SIF calculated for each species pair.

### Ethics statement

Jundiaí City Hall and private owners provided permission to conduct this project at Serra do Japi. During this research, the animals were observed in their natural environment and none of them were captured or handled. Therefore, there are no protocols to be reported to institutional or governmental agencies that regulate animal research.

## Results

### Spatial scale

Models with the same covariate measured at different extents (500 m vs. 1,000 m) were usually equally supported, with the exception of high-quality forest cover for ocelot, which was better explained by the 500 m extent than the 1,000 m extent (S5 Table).

### Single-species occupancy

**Fig 2:**
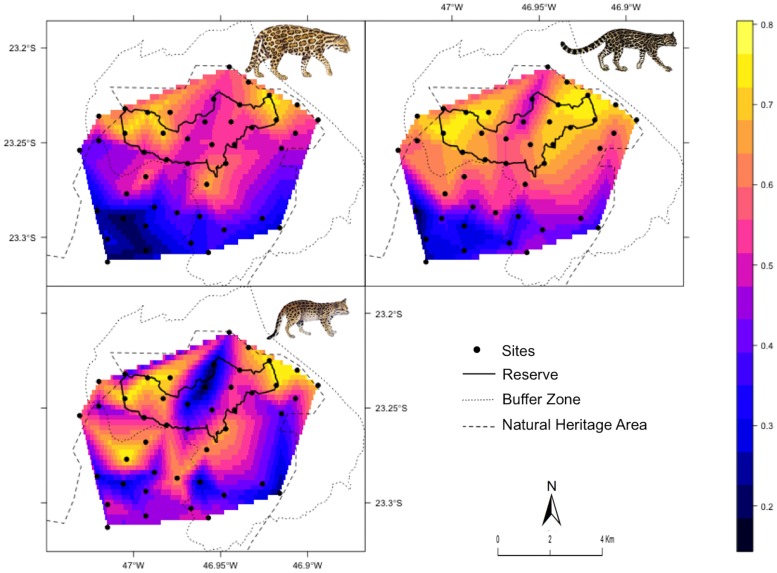
Neotropical spotted cats’ site occupancy. Interpolated site occupancy of the three spotted cats at an Atlantic Forest site in Brazil: ocelot—*Leopardus pardalis* (top left), margay—*L*. *wiedii* (top right), oncilla—*L*. *guttulus* (bottom left).

**Fig 3:**
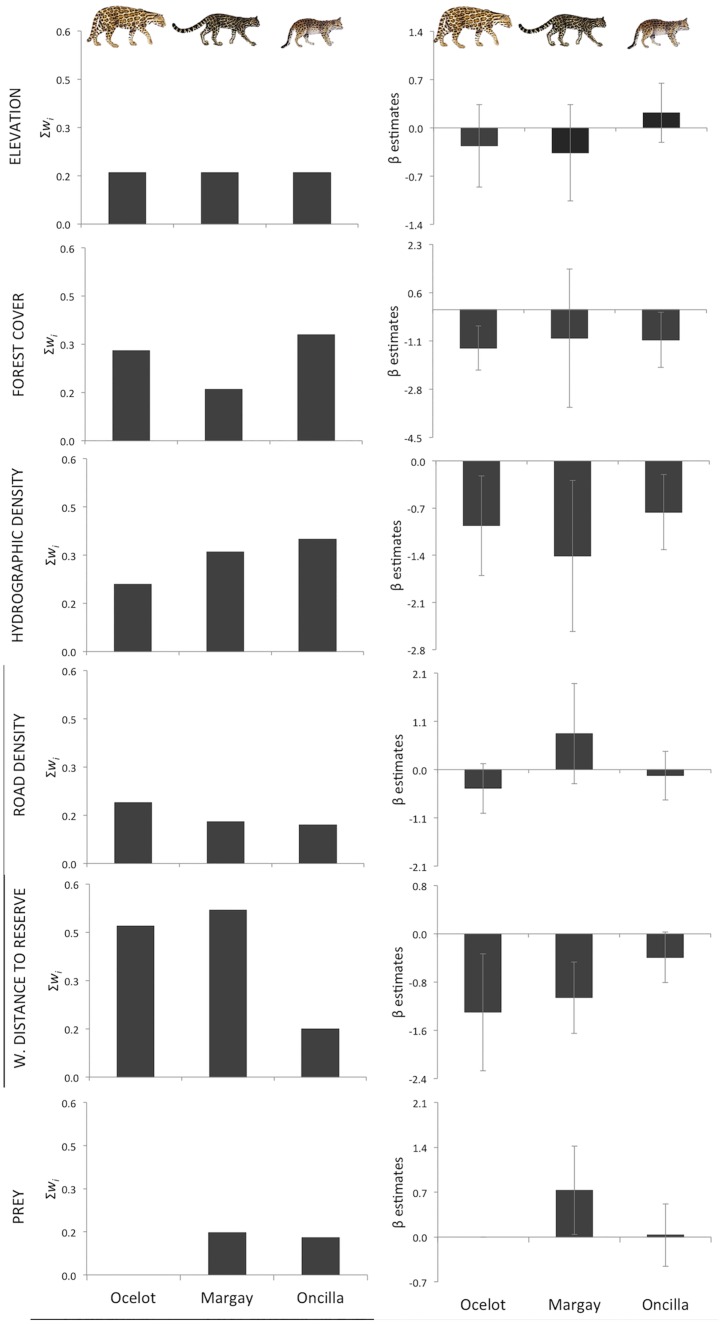
Covariates effect on the occupancy of Neotropical spotted cats. Influence of geomorphometry, environmental, and anthropogenic covariates on the occupancy of spotted cats in a large Atlantic Forest remnant, showing the sum of *w*_*i*_ (which indicates the relative importance of covariates) and the associated beta estimates with standard error estimated from the single-season single-species models.

We had 123 detections (N = 5,198 trap days) of the three Neotropical spotted cats (Table 1; S7 Table). We had some evidence that the habitat use of the cats varied according to landscape characteristics (Fig 2). Margay and ocelot had higher occupancies closer to a more protected area (i.e., reserve), and weighted distance to reserve border was the main factor influencing their habitat use, emerging as the top-ranked model with high relative importance (Fig 3; S6 Table). All other analyzed covariates had overall low relevance (Fig 3; S6 Table).

**Table 1:** Number of records (detections) by each method (scat sampling and camera trap), number of sampling sites with detections, naïve occupancy, estimated occupancy probability ($\hat{\Psi }$) from single-season single-species models, and relative increase above naïve occupancy when using estimates of the three Neotropical spotted cats in a large Atlantic Forest remnant.

	N detections		N sites w. detections	Naïve occup.	Detection probability (*p*)			Rel. increase above naïve occup. (%)^2^
	Scats	Camera traps			Scats	Camera traps	Occup. prob. $\hat{\Psi }$^1^	
Ocelot	8	16	12	0.27	0.07	0.08	0.48 (±0.15)	80
Margay	27	12	17	0.38	0.11	0.05	0.64 (±0.17)	69
Oncilla	33	29	21	0.47	0.14	0.07	0.64 (±0.13)	37

^1^Occupancy probability and standard deviation estimated by model averaging.

^2^Percentage increase in estimated proportion of occupied sites when incorporating detection probability (*p*) [(estimated occupancy probability/naïve occupancy)-1*100].

Margay and oncilla, the two smaller and more cryptic cats, had more detections from scat sampling, a method that had a detection probability of twice the detection probability of camera traps (Table 1).

### Co-occurrence patterns

There was no evidence that one species affects the occupancy of the other (Tables 2 and 3). For all three pairs of species (ocelot-margay, ocelot-oncilla, and margay-oncilla), the models where the pair had similar occupancies and the subordinate species had similar occupancy regardless of the dominant being at the sampling site or not (i.e., ψA = ψBA = ψBa) were ranked as the top models (Table 2). Furthermore, models where the covariate ‘weighted distance to reserve border’ was incorporated with ocelot and margay occupancy were always ranked better than the models without this covariate, reinforcing the importance of the reserve for both species (Table 2).

**Table 2:** Co-occurrence occupancy models used to evaluate the role of interspecific interactions on the habitat use of three sympatric Neotropical spotted cats in a large Atlantic Forest remnant.

Model	AIC	ΔAIC	*w*_*i*_	K	-2LL
**Ocelot-Margay**					
ψA(reserve dist) = ψBA(reserve dist) = ψBa(reserve dist) *p*(global^1^)	349.56	0	0.76	9	326.42
ψA(reserve dist)≠ψBA(reserve dist) = ψBa(reserve dist) *p*(global)	352.25	2.69	0.20	10	325.78
ψA = ψBA = ψBa *p*(global)	355.87	6.31	0.03	8	335.87
ψA≠ψBA = ψBa *p*(global)	358.03	8.47	0.01	9	334.89
ψA≠ψBA≠ψBa *p*(global)	361.32	11.76	0.00	10	334.85
**Ocelot-Oncilla**					
ψA(reserve dist) = ψBA = ψBa *p*(global^2^)	386.13	0	0.34	8	366.13
ψA(reserve dist)≠ψBA = ψBa *p*(global)	389.20	3.07	0.07	9	366.06
ψA(reserve dist)≠ψBA≠ψBa *p*(global)	392.53	6.40	0.01	10	366.06
**Margay-Oncilla**					
ψA(reserve dist) = ψBA = ψBa *p*(global^3^)	443.56	0	0.60	5	432.02
ψA(reserve dist)≠ψBA = ψBa *p*(global)	445.84	2.28	0.19	6	431.63
ψA(reserve dist)≠ψBA≠ψBa *p*(global)	447.44	3.88	0.09	7	430.41
ψA,ψBA = ψBa *p*(global)	447.61	4.05	0.08	5	436.07
ψA≠ψBA≠ψBa *p*(global)	448.84	5.28	0.04	6	434.63
ψA = ψBA = ψBa *p*(global)	452.62	9.06	0.01	4	443.62

*p*(general^1^) = *p*A≠*r*A≠*p*B≠*r*BA≠*r*Ba; method;

*p*(general^2^) = *p*A = *r*A≠*p*B≠*r*BA≠*r*Ba; method;

*p*(general^3^) = *p*A = *r*A = *p*B = *r*BA = *r*Ba; method.

ψA = occupancy of dominant species; ψBA = occupancy of subordinate species when the dominant species is present; ψBa = occupancy of subordinate species when the dominant species is absent. Reserve dist = Covariate ‘weighted distance to reserve border’. We used “=” to designate that two or more parameters were set as equal (e.g., ψBA = ψBa means that the occupancy of the subordinate species is independent of that of the dominant species). We used “≠” to designate that two or more parameters were set as different (e.g., ψBA≠ψBa models assumed that the occupancy of the subordinate species was influenced by the dominant species).

**Table 3:** Occupancy (ψ), detection probability (*p* and *r*), and species interaction factor (SIF—phi and delta) estimated from co-occurrence occupancy models of three sympatric Neotropical spotted cats in a large Atlantic Forest remnant.

	ψA	ψBA	ψBa	*r*A	*p*A	*p*B	*r*BA	*r*Ba	Phi	Delta
Ocelot—Margay	0.60	0.63	0.63	0.06	0.06	0.08	0.08	0.08	1.00	1.00
Ocelot—Oncilla	0.67	0.80	0.80	0.05	0.05	0.04	0.41	0.10	1.00	3.53
Margay—Oncilla	0.61	0.68	0.63	0.09	0.08	0.08	0.13	0.12	1.03	1.11

ψA = occupancy of dominant species; ψBA = occupancy of subordinate species when the dominant species is present; ψBa = occupancy of subordinate species when the dominant species is absent; *r*A = probability of dominant species being detected when the subordinate species is present; *p*A = probability of dominant species being detected when the subordinate species is absent; *p*B = probability of subordinate species being detected when the dominant species is not present; *r*BA = probability of subordinate species being detected when the dominant species is present and detected; *r*Ba = probability of subordinate species being detected when the dominant species is present but not detected; Phi = ratio of how much more (>1) or less (<1) likely the species are to co-occur at a site compared to what would be expected if the species occurred independently of each other; Delta = ratio of how much more (>1) or less (<1) likely the species are to be detected together in a survey compared to what would be expected if they were detected independently.

We also found no evidence that the presence of margay or oncilla have an effect on the detection probability of the more dominant species, the ocelot (i.e., that *p*A≠*r*A), or that the presence or detection of ocelot has an effect on the detection of margay (i.e., that *p*B≠*r*BA = *r*Ba or *p*B≠*r*BA≠*r*Ba; Tables 3 and 4). However, we found evidence that the presence of margay and the presence and detection of ocelot increases the detection probability of oncilla (i.e., margay-oncilla: *p*B≠*r*BA = *r*Ba; ocelot-oncilla: *p*B≠*r*BA≠*r*Ba; Tables 3 and 4).

**Table 4:** Co-occurrence occupancy models used to evaluate the role of interspecific interactions on the detection probability of three sympatric Neotropical spotted cats in a large Atlantic Forest remnant.

Model	AIC	ΔAIC	*w*_*i*_	K	-2LL
**Ocelot-Margay**					
ψ(top) *p*A = *r*A = *p*B = rBA = rBa	339.14	0	0.77	5	327.60
ψ(top) *p*A = *r*A,*p*B = rBA = rBa	341.81	2.67	0.20	6	327.60
ψ(top) *p*A≠*r*A≠*p*B≠rBA = rBa	346.45	7.31	0.02	8	326.45
ψ(top) *p*A≠*r*A≠*p*B≠rBA≠rBa	349.56	10.42	0.00	9	326.42
**Ocelot-Oncilla**					
ψ(top) *p*A = *r*A≠*p*B≠rBA≠rBa	386.13	0	0.21	8	366.13
ψ(top) *p*A = *r*A = *p*B = rBA = rBa	391.69	5.56	0.01	5	380.15
ψ(top) *p*A≠*r*A≠*p*B = rBA = rBa	393.20	7.07	0.01	7	376.17
ψ(top) *p*A = *r*A≠*p*B = rBA = rBa	394.36	8.23	0.00	6	380.15
**Margay-Oncilla**					
ψ(top) *p*A = *r*A = *p*B = rBA = rBa	443.56	0	0.28	5	432.02
ψ(top) *p*A = *r*A≠*p*B≠rBA = rBa	444.05	0.49	0.22	7	427.02
ψ(top) *p*A = *r*A≠*p*B = rBA = rBa	444.96	1.40	0.14	6	430.75
ψ(top) *p*A≠*r*A≠*p*B≠rBA = rBa	445.41	1.85	0.11	8	425.41
ψ(top) *p*A≠*r*A≠*p*B = rBA = rBa	445.46	1.90	0.11	7	428.43
ψ(top) *p*A = *r*A≠*p*B≠rBA≠rBa	445.69	2.13	0.10	8	425.69
ψ(top) *p*A≠*r*A≠*p*B≠rBA≠rBa	447.42	3.86	0.04	9	424.28

While modeling detection probability (*p*) for each pair of species, we incorporated the covariate “method” in *p* and kept occupancy (ψ) as it was in the top-ranked model from the co-occurrence occupancy models used to evaluate ψ (Table 2): Ocelot vs. Margay ψ(top) = [ψA(reserve dist) = ψBA(reserve dist) = ψBa(reserve dist)]; Ocelot vs. Oncilla ψ(top) = [ψA(reserve dist)≠ψBA = ψBa]; Margay vs. Oncilla ψ(top) = ψA = ψBA = ψBa.

## Discussion

When investigating species’ distribution and use of habitat, assessing the relative importance of landscape attributes and the role of interspecific interactions is often difficult. Here, we used occupancy modeling [41, 42] to explicitly incorporate detection probability and habitat variables while examining co-occurrence patterns and landscape use by three sympatric and morphologically similar species of Neotropical cats. Predators can be considered a key group because they affect prey and plant populations, influencing ecosystem dynamics [1–3]. However, most species of felids are threatened, or we lack basic information about them. All three Neotropical spotted cats analyzed here are believed to be suffering from population decrease, and two (*L*. *wiedii* and *L*. *guttulus*) are considered ‘Near Threatened’ or ‘Vulnerable’ [96]. We demonstrated that the proximity to a more protected area (nature reserve) is the main factor influencing the habitat use of Neotropical spotted cats within their home ranges in a large Atlantic forest remnant, and we had low support for the hypothesis that interspecific interactions modulate how they use the landscape.

Our first prediction that among the landscape characteristics, the human-related variables would be more important predictors of landscape use by Neotropical spotted cats was in part corroborated. Roads and human accessibility, for instance, are important determinants of the occupancy of sensitive species (e.g., game species—[97]); and road kills can be common among felids and other carnivores [98, 99]. Here, we had some evidence that road density, which is also a measure of human accessibility, may have a negative effect on occupancy of some spotted cats (given its negative beta estimates for two out of the three species analyzed), particularly on ocelots; however, it did not have a strong effect and was not a major factor for them. Besides area accessibility, higher protection status is also usually associated with lower human-related pressures [9, 17, 18]. Our results showed that more strict protection status is important for the Neotropical spotted cats, especially for ocelots and margays, which used more areas closer to the reserve even though our entire study area is under some type of protection. Although ‘weighted distance to reserve border’ was not a high-ranked model for oncilla, the influence of this covariate on its occupancy was also in the predicted direction (negative). In previous studies, only ocelots or larger felids (pumas and jaguars) were more commom in areas with higher legal protection than in less protected areas [100, 101]. Whether these distinct outcomes are derived from differences in the felid community structure (e.g., more larger felids occuring in these previous studies than in our study area) or arise from some intrinsic local characteristic of the sites (e.g., level of anthropogenic pressures) can only be answered with further studies.

Prey availability can usually influence the abundance, density, occupancy and habitat use of carnivores [56, 102–105]. Although the relationship between the prey index and occupancy was in the predicted direction (positive), we did not have enough evidence that this variable, hydrographic density, high-quality forest cover, and elevation influence the spotted cats’ habitat use. The possible opportunistic feeding behavior of Neotropical small cats [36] could explain the lack of effect of prey on their habitat use. However, we also note that prey availability, measured as the sum of prey occupancy at each sampling site, varied only slightly across sites (mean 1.31±0.12) and did not account for differences in prey density. Therefore, we cannot yet discard a possible effect of prey on spotted cats’ habitat use.

Intraguild competition can be an important determinant of carnivore abundance and distribution, as it can lead to spatial or temporal segregation among species [7, 28, 106]. However, when examining species co-occurrence patterns, it is often difficult to distinguish the difference between habitat preferences and competitive exclusions. Furthermore, since species present at a location are not always detected with certainty, incorporating detection probability along with habitat preferences directly into the model set may avoid incorrect inferences about co-occurrence patterns [41]. By adopting this approach, we found no evidence that the presence of either ocelot, margay, or oncilla have a negative influence on how the other species use the habitat. Thus competitive exclusion among them is unlikely, at least within the analyzed scale, and at a relatively conserved and protected area such as ours. The lack of spatial partitioning based on interspecific interaction was also found for North American carnivores, and similarly to our study, habitat preferences were more important in structuring the community [107, 108]. Nonetheless, because occupancy does not account for variations in density, it is still possible that the presence of one species affects the density of the other, as previously suggested [109]. We also encourage further research to analyze the effects of larger felids (e.g., pumas and jaguars) on the habitat use of smaller cats, which unfortunately, we did not have data to investigate (only six detections of pumas and none of jaguar). In addition, since land use and human activity can alter occupancy patterns [110] and behavior [111] of carnivores, it would be of interest to explore whether interspecific relations and co-occurrence patterns among small felids are affected by different degrees of human disturbances, which unfortunately we did not have enough data to investigate. Finally, although the models used here can yield strong inferences about species co-occurrence patterns, this does not imply strong inference about the processes that generated the observed patterns, and it would be useful to observe the system dynamics over time [41].

We found evidence that one species affects the behavior of the other in at least one respect, as oncilla is more likely to be detected if margay is present or if ocelot is either present or detected. Felids are highly territorial and use scent marks such as urine and feces to mark their territories [62]. Two things should be noted from the fact that most of the felids’ detections came from scat sampling, and therefore, their territorial scent mark: first, since we collected the feces as they were detected, sites with scat detections became unmarked after each sampling occasion. Second, scent marks are frequently over-marked either by the same animal or other individuals [62]. Therefore, we suggest that the presence of a spotted cat (and consequently, its feces) increased the detection of another spotted cat as it was attracted to either over-mark the feces (before it was removed) or use an area rendered unmarked (through collection of the scats). The small felids may be able to avoid direct confrontations by marking recently used areas within their home range and using different parts of their home range according to where the other is currently marking (i.e., using the area). However, to clarify the mechanisms underlying our findings, more detailed studies on the behavior of the Neotropical spotted cats are necessary. What we can suggest so far is that territorial demarcation may potentially help to regulate how these small felids share their habitats, consequently alleviating or reducing the competition among them.

In conclusion, our results suggest that human-related factors, such as distance to a reserve, are more important drivers of Neotropical spotted cats’ habitat use than are interspecific interactions, environmental landscape attributes, geomorphometry or, potentially, prey availability. We suggest that the dietary overlap of the three species might be small enough to allow co-existence [57–61], or that another behavioral mechanism in addition to differences in habitat preference, such as time partitioning, may allow them to co-exist [100, 112, 113].

### Management recommendations

Given the importance and vulnerability of spotted cats in Neotropical forests, several actions are required for their conservation, including enhancing forest connectivity and gathering basic information on their ecology and behavior [4, 114]. Here we underline the main management recommendations resulting from our study:

1. We demonstrated that the most important factor for managing small felids can be the maintenance and establishment of nature reserves and other areas that are managed to ensure minimal disturbance and human presence (i.e., IUCN Protected Area Category Ia—Strict Nature Reserve [115]). Protected areas can decrease habitat loss [116] and anthropogenic pressures [17, 18] as well as improve the occupancy of key groups (Nagy-Reis et al. present study). However, because poaching may still occur illegally, effective law enforcement and other management actions such as environmental education are also important to ensure the conservation of small felids.
2. The use of methods that incorporate detection probability is critical to the understanding of species’ responses to habitat and interspecific interactions. We showed how dealing with imperfect detection results in a substantial relative increase above naïve occupancy for rare, cryptic, and elusive animals such as small felids, reinforcing the importance of accounting for detection probability in ecological and behavioral studies on such species. Our research also emphasizes the feasibility of alternative methods for surveying felids, such as scat sampling combined with tricology, which has a higher detection probability than camera traps and supplies non-invasive material for studying diet and performing molecular analysis, providing essential information on population parameters and ecology [63, 117]. We also confirmed the reliability of tricology as a low cost alternative to molecular methods for identification of Neotropical felids, as long as meticulous procedure is adopted by a trained researcher.

## Supporting Information

S1 Fig Location of the sampling sites at the study area (Serra do Japi, Brazil) where Neotropical spotted cats were sampled using camera trap and scat sampling. Sampling sites represented with a same color were sampled simultaneously within each campaign. Campaign 1 (April 2013 to September 2013)–Group A (black): Apr-Mai; Group B (blue): Jun-Jul; Group C (green): Ago-Sep. Campaign 2 (October 2013 to March 2014)–Group A: Oct-Nov; Group B: Dec-Jan; Group C: Feb-Mar. Campaign 3 (April 2014 to September 2014)–Group A: Apr-Mai; Group B: Jun-Jul; Group C: Ago-Sep.
(PDF)

S2 Fig Photos of the cuticular impressions of each Neotropical spotted cat at 400x magnification. From the left to the right: ocelot (*Leopardus pardalis*), margay (*L*. *wiedii*), and oncilla (*L*. *guttulus*).
(PDF)

S1 Table Spatial independence of detections for three Neotropical spotted cats sampled with camera trap and scat sampling (1.5 km between sampling sites) at a large Atlantic Forest remnant in Brazil. Results of Moran’s *I* autocorrelation tests [1]—based on the number of detection records of each species and the geographic position of each site. We used R 2.13.0 software [2] and the *ape* package [3]. 1. Legendre P & Legendre L. Numerical Ecology. New York: Elsevier; 1998. 2. R Development Core Team. R: A language and environment for statistical computing. R Foundation for Statistical Computing. 2014. Available: <www.R-project.org>. Accessed 2 Oct 2014. 3. Paradis E, Blomberg S, Boljer B, Claude J, Cuong HS. et al. Analyses of phylogenetics and evolution: package “ape”. Available: <ape-package.ird.fr>. Accessed 10 Jul 2015.
(PDF)

S2 Table Spearman’s correlation matrix for the site covariates measured at a large Atlantic Forest remnant in Brazil. The four landscape site covariates (elevation, hydrographic density, road density and percentage of high-quality forest cover) were measured at 500 and 1,000 m spatial extents (buffer sizes). Highly correlated (r_s_<0.50) outcomes are in bold. S prey = prey index for margay and oncilla; O prey = prey index for ocelot; * = excluded covariates.
(PDF)

S3 Table Multi-season single-species occupancy models used to evaluate the effect of time (“campaign”) on the habitat use of sympatric Neotropical spotted cats at a large Atlantic Forest remnant in Brazil. *p*(general) = campaign + method + soil coverage + percentage of high-quality forest cover at 500 m buffer size; “gamma” = colonization; “eps” = extinction; “.” = no covariate included; “0” = parameter was fixed to 0.
(PDF)

S4 Table Model selection analysis for detection probability (*p*) covariates for three Neotropical spotted cats at a large Atlantic Forest remnant in Brazil. “Campaign” = campaign in which data was collected (1- April 2013 to September 2013; 2- October 2013 to March 2014; 3- April 2014 to September 2014); “Groups” = order within the campaigns that sampling sites were surveyed; “Method” = method used in each sampling occasion (camera trapping or scat sampling); “.” = no covariate included (i.e., null model).
(PDF)

S5 Table Model selection analysis for occupancy (ψ) covariates (elevation, percentage of high-quality forest cover, hydrographic density and road density) measured at different spatial extents (buffer sizes) for three Neotropical spotted cats at a large Atlantic Forest remnant in Brazil. *p*(general) = method + soil coverage + percentage of high-quality forest cover at 500 m buffer size.
(PDF)

S6 Table Single-season single-species occupancy models (cumulative *w*_*i*_ >0.80) used to evaluate the effect of weighted distance to reserve border, geomorphometry, environmental, and anthropogenic landscape attributes on the habitat use of sympatric Neotropical spotted cats at a large Atlantic Forest remnant in Brazil. *p*(general) = method + soil coverage + percentage of high-quality forest cover at 500 m buffer size.
(PDF)

S7 Table Spearman’s correlation matrix for the three Neotropical spotted cats found at a large Atlantic Forest remnant in Brazil (based on the number of detection records at each sampling site). (PDF)
