# Supplementary material for: Landscape Use and Co-Occurrence Patterns of Neotropical Spotted Cats
Source: PLoS One. 2017 Jan 4;12(1):e0168441. doi: 10.1371/journal.pone.0168441 (PMC5215768; doi:10.1371/journal.pone.0168441)
Supplement: S1 Fig — Sampling sites represented with a same color were sampled simultaneously within each campaign. Campaign 1 (April 2013 to September 2013)–Group A (black): Apr-Mai; Group B (blue): Jun-Jul; Group C (green): Ago-Sep. Campaign 2 (October 2013 to March 2014)–Group A: Oct-Nov; Group B: Dec-Jan; Group C: Feb-Mar. Campaign 3 (April 2014 to September 2014)–Group A: Apr-Mai; Group B: Jun-Jul; Group C: Ago-Sep. (PDF) [file pone.0168441.s001.pdf]

S1 Figure. Location of the sampling sites at the study area (Serra do Japi, Brazil) where Neotropical spotted cats were sampled using camera trap and scat sampling.

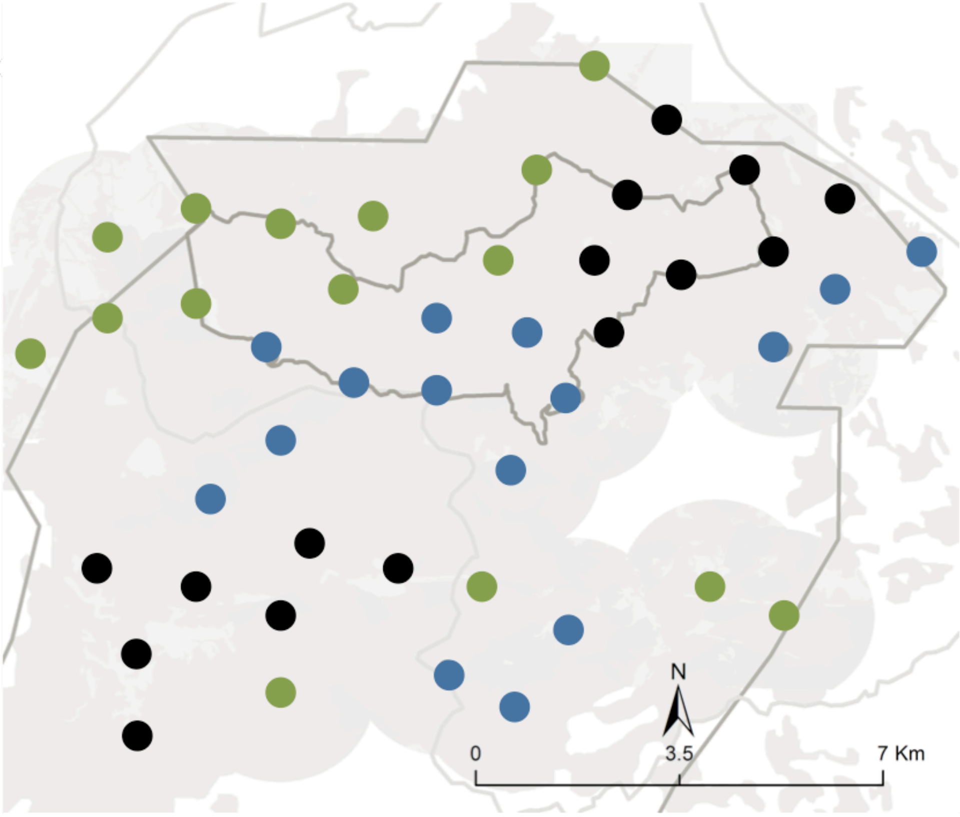

Sampling sites represented with a same color were sampled simultaneously within each campaign. Campaign 1 (April 2013 to September 2013) – Group A (black): Apr-Mai; Group B (blue): Jun-Jul; Group C (green): Ago-Sep. Campaign 2 (October 2013 to March 2014) – Group A: Oct-Nov; Group B: Dec-Jan; Group C: Feb-Mar. Campaign 3 (April 2014 to September 2014) – Group A: Apr-Mai; Group B: Jun-Jul; Group C: Ago-Sep.
