# Supplementary material for: Landscape Use and Co-Occurrence Patterns of Neotropical Spotted Cats
Source: PLoS One. 2017 Jan 4;12(1):e0168441. doi: 10.1371/journal.pone.0168441 (PMC5215768; doi:10.1371/journal.pone.0168441)
Supplement: S2 Fig — From the left to the right: ocelot (Leopardus pardalis), margay (L. wiedii), and oncilla (L. guttulus). (PDF) [file pone.0168441.s002.pdf]

S2 Figure. Photos of the cuticular impressions of each Neotropical spotted cat at 400x magnification.

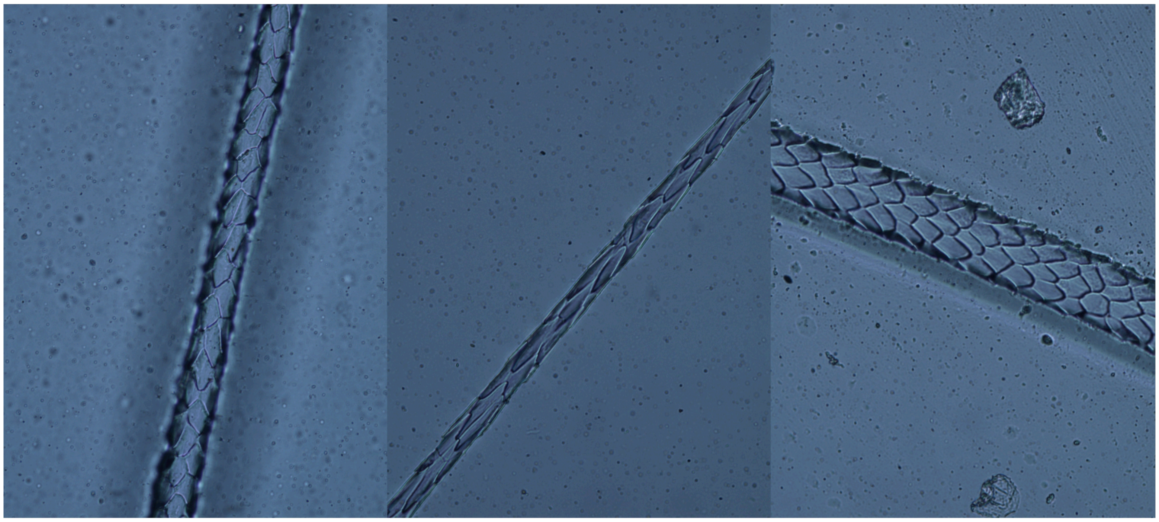

From the left to the right: ocelot (*Leopardus pardalis*), margay (*L. wiedii*), and oncilla (*L. guttulus*).
