# Supplementary material for: Landscape Use and Co-Occurrence Patterns of Neotropical Spotted Cats
Source: PLoS One. 2017 Jan 4;12(1):e0168441. doi: 10.1371/journal.pone.0168441 (PMC5215768; doi:10.1371/journal.pone.0168441)
Supplement: S1 Table — Results of Moran’s I autocorrelation tests [1]—based on the number of detection records of each species and the geographic position of each site. We used R 2.13.0 software [2] and the ape package [3]. 1. Legendre P & Legendre L. Numerical Ecology. New York: Elsevier; 1998. 2. R Development Core Team. R: A language and environment for statistical computing. R Foundation for Statistical Computing. 2014. Available: <www.R-project.org>. Accessed 2 Oct 2014. 3. Paradis E, Blomberg S, Boljer B, Claude J, Cuong HS. et al. Analyses of phylogenetics and evolution: package “ape”. Available: <ape-package.ird.fr>. Accessed 10 Jul 2015. (PDF) [file pone.0168441.s003.pdf]

**Nagy-Reis, M.B.; Nichols, J.D.; Chiarello, A.G.; Ribeiro, M.C.; Setz, E.Z.F. Landscape Use and Co-occurrence Patterns of Neotropical Spotted Cats -** Supporting Information

S1 Table. Spatial independence of detections for three Neotropical spotted cats sampled with camera trap and scat sampling (1.5 km between sampling sites) at a large Atlantic Forest remnant in Brazil.

| | E(I) | I | P |
|---|---|---|---|
| Ocelot | -0.02 | -0.03 | 0.60 |
| Margay | -0.02 | -0.01 | 0.37 |
| Oncilla | -0.02 | -0.05 | 0.12 |

Results of Moran's *I* autocorrelation tests [1] - based on the number of detection records of each species and the geographic position of each site. We used R 2.13.0 software [2] and the *ape* package [3]. 1. Legendre P & Legendre L. Numerical Ecology. New York: Elsevier; 1998. 2. R Development Core Team. R: A language and environment for statistical computing. R Foundation for Statistical Computing. 2014. Available: <www.R-project.org>. Accessed 2 Oct 2014. 3. Paradis E, Blomberg S, Boljer B, Claude J, Cuong HS. et al. Analyses of phylogenetics and evolution: package "ape". Available: <ape-package.ird.fr>. Accessed 10 Jul 2015.
