# Supplementary material for: Landscape Use and Co-Occurrence Patterns of Neotropical Spotted Cats
Source: PLoS One. 2017 Jan 4;12(1):e0168441. doi: 10.1371/journal.pone.0168441 (PMC5215768; doi:10.1371/journal.pone.0168441)
Supplement: S2 Table — The four landscape site covariates (elevation, hydrographic density, road density and percentage of high-quality forest cover) were measured at 500 and 1,000 m spatial extents (buffer sizes). Highly correlated (rs<0.50) outcomes are in bold. S prey = prey index for margay and oncilla; O prey = prey index for ocelot; * = excluded covariates. (PDF) [file pone.0168441.s004.pdf]

Nagy-Reis, M.B.; Nichols, J.D.; Chiarello, A.G.; Ribeiro, M.C.; Setz, E.Z.F. Landscape Use and Co-occurrence Patterns of Neotropical Spotted Cats - Supporting Information

S2 Table. Spearman's correlation matrix for the site covariates measured at a large Atlantic Forest remnant in Brazil.

	Elev 500	Elev 1000	Hydro 500	Hydro 1000	Roads 500	Roads 1000	Veg 500	Veg 1000*	S prey	O prey*
Elev500	-	-	-0.07	0.03	0.19	-0.13	0.25	**0.54**	0.21	**-0.82**
Elev1000	-	-	0.03	0.11	0.20	-0.12	0.27	**0.58**	0.20	**-0.81**
Hydro500			-	-	0.07	-0.12	0.23	0.18	0.06	0.26
Hydro1000			-	-	0.01	-0.24	0.34	0.26	-0.01	0.27
Roads500					-	-	-0.20	-0.14	0.35	0.33
Roads1000					-	-	-0.35	-0.39	0.33	0.23
Veg500							-	-	-0.25	-0.26
Veg1000							-	-	-0.16	-0.04
S prey									-	-
Ocelot prey									-	-

The four landscape site covariates (elevation, hydrographic density, road density and percentage of high-quality forest cover) were measured at 500 and 1,000 m spatial scales (buffer sizes). Highly correlated ($r<0.50$) outcomes are in bold. S prey = prey index for margay and oncilla; O prey = prey index for ocelot; * = excluded covariates.
