# Supplementary material for: Landscape Use and Co-Occurrence Patterns of Neotropical Spotted Cats
Source: PLoS One. 2017 Jan 4;12(1):e0168441. doi: 10.1371/journal.pone.0168441 (PMC5215768; doi:10.1371/journal.pone.0168441)
Supplement: S3 Table — p(general) = campaign + method + soil coverage + percentage of high-quality forest cover at 500 m buffer size; “gamma” = colonization; “eps” = extinction; “.” = no covariate included; “0” = parameter was fixed to 0. (PDF) [file pone.0168441.s005.pdf]

Nagy-Reis, M.B.; Nichols, J.D.; Chiarello, A.G.; Ribeiro, M.C.; Setz, E.Z.F. Landscape Use and Co-occurrence Patterns of Neotropical Spotted Cats - Supporting Information

S3 Table. Multi-season single-species occupancy models used to evaluate the effect of time ("campaign") on the habitat use of sympatric Neotropical spotted cats at a large Atlantic Forest remnant in Brazil.

Model	AICc	ΔAIC	w_i	K	-2LL
**Ocelot**					
ψ(.) gamma(0) eps(0) *p*(general)	163.37	0	0.97	10	136.9
ψ(.) gamma(.) eps(.) *p*(general)	170.48	7.11	0.03	12	136.73
**Margay**					
ψ(.) gamma(0) eps(0) *p*(general)	205.03	0	0.92	8	185.03
ψ(.) gamma(.) eps(.) *p*(general)	210.01	4.98	0.08	10	183.54
**Oncilla**					
ψ(.) gamma(0) eps(0) *p*(general)	256.02	0	0.97	9	232.88
ψ(.) gamma(.) eps(.) *p*(general)	262.87	6.85	0.03	11	232.87

p(general) = campaign + method + soil coverage + percentage of high-quality forest cover at 500 m buffer size; "gamma" = colonization; "eps" = extinction; "." = no covariate included; "0" = parameter was fixed to 0.
