# Supplementary material for: Landscape Use and Co-Occurrence Patterns of Neotropical Spotted Cats
Source: PLoS One. 2017 Jan 4;12(1):e0168441. doi: 10.1371/journal.pone.0168441 (PMC5215768; doi:10.1371/journal.pone.0168441)
Supplement: S4 Table — “Campaign” = campaign in which data was collected (1- April 2013 to September 2013; 2- October 2013 to March 2014; 3- April 2014 to September 2014); “Groups” = order within the campaigns that sampling sites were surveyed; “Method” = method used in each sampling occasion (camera trapping or scat sampling); “.” = no covariate included (i.e., null model). (PDF) [file pone.0168441.s006.pdf]

Nagy-Reis, M.B.; Nichols, J.D.; Chiarello, A.G.; Ribeiro, M.C.; Setz, E.Z.F. Landscape Use and Co-occurrence Patterns of Neotropical Spotted Cats - Supporting Information

S4 Table. Model selection analysis for detection probability (*p*) covariates for three Neotropical spotted cats at a large Atlantic Forest remnant in Brazil.

Model	AICc	ΔAIC	w_i	K	-2LL
**Ocelot**					
ψ(.) *p*(.)	148.14	0	0.48	2	143.85
ψ(.) *p*(campaign)	149.06	0.92	0.30	4	140.06
ψ(.) *p*(method)	150.37	2.23	0.16	3	143.78
ψ(.) *p*(groups)	152.07	3.93	0.07	4	143.07
**Margay**					
ψ(.) *p*(method)	197.68	0	0.75	3	191.09
ψ(.) *p*(.)	200.47	2.79	0.19	2	196.18
ψ(.) *p*(groups)	203.53	5.85	0.04	4	194.53
ψ(.) *p*(campaign)	204.47	6.79	0.03	4	195.47
**Oncilla**					
ψ(.) *p*(method)	250.10	0	0.50	3	243.51
ψ(.) *p*(campaign)	251.23	1.13	0.29	4	242.23
ψ(.) *p*(.)	252.09	1.99	0.19	2	247.80
ψ(.) *p*(groups)	256.27	6.17	0.02	4	247.27

"Campaign" = campaign in which data was collected (1- April 2013 to September 2013; 2- October 2013 to March 2014; 3- April 2014 to September 2014); "Groups" = order within the campaigns that sampling sites were surveyed; "Method" = method used in each sampling occasion (camera trapping or scat sampling); "." = no covariate included (i.e., null model).
