# Supplementary material for: Landscape Use and Co-Occurrence Patterns of Neotropical Spotted Cats
Source: PLoS One. 2017 Jan 4;12(1):e0168441. doi: 10.1371/journal.pone.0168441 (PMC5215768; doi:10.1371/journal.pone.0168441)
Supplement: S5 Table — p(general) = method + soil coverage + percentage of high-quality forest cover at 500 m buffer size. (PDF) [file pone.0168441.s007.pdf]

Nagy-Reis, M.B.; Nichols, J.D.; Chiarello, A.G.; Ribeiro, M.C.; Setz, E.Z.F. Landscape Use and Co-occurrence Patterns of Neotropical Spotted Cats - Supporting Information

S5 Table. Model selection analysis for occupancy (ψ) covariates (elevation, percentage of high-quality forest cover, hydrographic density and road density) measured at different spatial scales (buffer sizes) for three Neotropical spotted cats at a large Atlantic Forest remnant in Brazil.

Model	AICc	ΔAICc	w_i	K	-2LL
ELEVATION					
**Ocelot**					
ψ(.) *p*(general)	154.78	0	0.64	5	143.24
ψ(elev500) *p*(general)	157.28	2.50	0.18	6	143.07
ψ(elev1000) *p*(general)	157.42	2.64	0.17	6	143.21
**Margay**					
ψ(.) *p*(general)	202.20	0	0.61	5	190.66
ψ(elev500) *p*(general)	204.30	2.10	0.21	6	190.09
ψ(elev1000) *p*(general)	204.71	2.51	0.17	6	190.50
**Oncilla**					
ψ(.) *p*(general)	252.75	0	0.62	5	241.21
ψ(elev500) *p*(general)	255.07	2.32	0.19	6	240.86
ψ(elev1000) *p*(general)	255.15	2.40	0.19	6	240.94
HIGH-QUALITY FOREST COVER					
**Ocelot**					
ψ(.) *p*(general)	154.78	0	0.45	5	143.24
ψ(forest500) *p*(general)	154.86	0.08	0.43	6	140.65
ψ(forest1000) *p*(general)	157.35	2.57	0.12	6	143.14
**Margay**					
ψ(.) *p*(general)	202.20	0	0.60	5	190.66
ψ(forest1000) *p*(general)	204.13	1.93	0.23	6	189.92
ψ(forest500) *p*(general)	204.65	2.45	0.18	6	190.44
**Oncilla**					
ψ(.) p(general)	252.75	0	0.58	5	241.21
ψ(forest500) p(general)	254.28	1.53	0.27	6	240.07
ψ(forest1000) p(general)	255.40	2.65	0.15	6	241.19
HYDROGRAPHIC DENSITY					
**Ocelot**					

ψ(.) *p*(general)	154.78	0	0.61	5	143.24
ψ(hydro1000) *p*(general)	157.01	2.23	0.20	6	142.80
ψ(hydro500) *p*(general)	157.11	2.33	0.19	6	142.90
**Margay**					
ψ(hydro1000) *p*(general)	201.69	0	0.44	6	187.48
ψ(.) *p*(general)	202.20	0.51	0.34	5	190.66
ψ(hydro500) *p*(general)	203.12	1.43	0.22	6	188.91
**Oncilla**					
ψ(.) *p*(general)	252.75	0	0.39	5	241.21
ψ(hydro1000) *p*(general)	253.08	0.33	0.33	6	238.87
ψ(hydro500) *p*(general)	253.35	0.60	0.29	6	239.14
ROADS DENSITY					
**Ocelot**					
ψ(.) *p*(general)	154.78	0	0.51	5	143.24
ψ(roads500) *p*(general)	155.86	1.08	0.30	6	141.65
ψ(roads1000) *p*(general)	156.82	2.04	0.19	6	142.61
**Margay**					
ψ(.) *p*(general)	202.20	0	0.56	5	190.66
ψ(roads1000) *p*(general)	203.49	1.29	0.29	6	189.28
ψ(roads500) *p*(general)	204.82	2.62	0.15	6	190.61
**Oncilla**					
ψ(.) *p*(general)	252.75	0	0.63	5	241.21
ψ(roads500) *p*(general)	255.02	2.27	0.20	6	240.81
ψ(roads1000) *p*(general)	255.37	2.62	0.17	6	241.16

p(general) = method + soil coverage + percentage of high-quality forest cover at 500 m buffer size.
