# Supplementary material for: Landscape Use and Co-Occurrence Patterns of Neotropical Spotted Cats
Source: PLoS One. 2017 Jan 4;12(1):e0168441. doi: 10.1371/journal.pone.0168441 (PMC5215768; doi:10.1371/journal.pone.0168441)
Supplement: S6 Table — p(general) = method + soil coverage + percentage of high-quality forest cover at 500 m buffer size. (PDF) [file pone.0168441.s008.pdf]

Nagy-Reis, M.B.; Nichols, J.D.; Chiarello, A.G.; Ribeiro, M.C.; Setz, E.Z.F. Landscape Use and Co-occurrence Patterns of Neotropical Spotted Cats - Supporting Information

S6 Table. Single-season single-species occupancy models (cumulative w_i>0.80) used to evaluate the effect of weighted distance to reserve border, geomorphometry, environmental, and anthropogenic landscape attributes on the habitat use of sympatric Neotropical spotted cats at a large Atlantic Forest remnant in Brazil.

					Beta estimates					
Model	ΔAICc	w_i	K	-2LL	Elevation	Forest cover	Hydrography	Roads	Reserve distance	Prey
**Ocelot**										
ψ(reserve dist) *p*(general)	0	0.25	6	139.03	-	-	-	-	-1.30 (±0.97)	-
ψ(.) *p*(general)	1.54	0.12	5	143.24	-	-	-	-	-	-
ψ(forest500) *p*(general)	1.62	0.11	6	140.65	-	-1.35 (±0.78)	-	-	-	-
ψ(reserve dist+roads1000) *p*(general)	2.12	0.09	7	138.33	-	-	-	-0.49 (±0.69)	-1.46 (±1.39)	-
ψ(forest500+hydro1000) *p*(general)	2.14	0.09	7	138.35	-	-1.82 (±0.92)	-0.96 (±0.74)	-	-	-
ψ(reserve dist+elev1000) *p*(general)	2.64	0.07	7	138.85	-0.26 (±0.60)	-	-	-	-1.38 (±0.95)	-
ψ(reserve dist+hydro1000) *p*(general)	2.77	0.06	7	138.98	-	-	-0.13 (±0.57)	-	-1.27 (±0.99)	-
ψ(roads1000) *p*(general)	3.58	0.04	6	142.61	-	-	-	-0.41 (±0.54)	-	-
**Margay**										

ψ(reserve dist) *p*(general)	0	0.18	6	186.39	-	-	-	-	-1.06 (±0.59)	-
ψ(reserve dist+hydro1000) *p*(general)	1.09	0.10	7	184.66	-	-	-1.09 (±1.08)	-	-0.90 (±0.58)	-
ψ(hydro1000) *p*(general)	1.09	0.10	6	187.48	-	-	-1.41 (±1.12)	-	-	-
ψ(.) *p*(general)	1.60	0.08	5	190.66	-	-	-	-	-	-
ψ(reserve dist+roads1000) *p*(general)	1.63	0.08	7	185.20	-	-	-	0.64 (±0.67)	-1.06 (±0.60)	-
ψ(reserve dist+forest1000) *p*(general)	2.11	0.06	7	185.68	-	-1.00 (±2.44)	-	-	-1.19 (±0.88)	-
ψ(reserve dist+elev1000) *p*(general)	2.50	0.05	7	186.07	-0.36 (±0.70)	-	-	-	-1.26 (±0.78)	-
ψ(reserve dist+prey) *p*(general)	2.73	0.05	7	186.30	-	-	-	-	-0.98 (±0.62)	0.16 (±0.54)
ψ(prey) *p*(general)	2.69	0.05	6	189.08	-	-	-	-	-	0.73 (±0.69)
ψ(hydro1000+elev1000) *p*(general)	2.78	0.04	7	186.35	0.68 (±0.75)	-	-1.98 (±1.64)	-	-	-
ψ(roads1000) *p*(general)	2.89	0.04	6	189.28	-	-	-	0.78 (±1.09)	-	-
**Oncilla**										
ψ(forest500+elev1000) *p*(general)	0	0.15	7	235.60	1.72 (±0.93)	-3.20 (±0.75)	-	-	-	-
ψ(.) *p*(general)	0.12	0.14	5	241.21	-	-	-	-	-	-
ψ(hydro1000) *p*(general)	0.45	0.12	6	238.87	-	-	-0.76 (±0.56)	-	-	-
ψ(hydro1000+elev1000) *p*(general)	1.37	0.07	7	236.97	1.29 (±1.26)	-	-2.14 (±2.20)	-	-	-
ψ(forest500) *p*(general)	1.65	0.06	6	240.07	-	-1.06 (±0.97)	-	-	-	-
ψ(hydro1000+forest500) *p*(general)	1.79	0.06	7	237.39	-	-0.85 (±0.58)	-1.10 (±0.85)	-	-	-
ψ(reserve dist) *p*(general)	1.94	0.06	6	240.36	-	-	-	-	-0.39 (±0.42)	-

ψ(hydro1000+prey) *p*(general)	2.50	0.04	7	238.10	-	-	-1.05 (±0.73)	-	-	-0.45 (±0.51)
ψ(elev1000) *p*(general)	2.52	0.04	6	240.94	0.22 (±0.43)	-	-	-	-	-
ψ(roads1000) *p*(general)	2.74	0.04	6	241.16	-	-	-	-0.13 (±0.53)	-	-
ψ(prey) *p*(general)	2.79	0.04	6	241.21	-	-	-	-	-	0.03 (±0.49)

p(general) = method + soil coverage + percentage of high-quality forest cover at 500 m buffer size.
