# Supplementary material for: Landscape Use and Co-Occurrence Patterns of Neotropical Spotted Cats
Source: PLoS One. 2017 Jan 4;12(1):e0168441. doi: 10.1371/journal.pone.0168441 (PMC5215768; doi:10.1371/journal.pone.0168441)
Supplement: S7 Table — (PDF) [file pone.0168441.s009.pdf]

Nagy-Reis, M.B.; Nichols, J.D.; Chiarello, A.G.; Ribeiro, M.C.; Setz, E.Z.F. Landscape Use and Co-occurrence Patterns of Neotropical Spotted Cats - Supporting Information

S7 Table. Spearman's correlation matrix for the three Neotropical spotted cats found at a large Atlantic Forest remnant in Brazil (based on the number of detection records at each sampling site).

	Ocelot	Margay	Oncilla
Ocelot	-	0.08	0.01
Margay		-	0.41
Oncilla			-
